# Supplementary material for: Spanish HCMV Seroprevalence in the 21st Century
Source: Viruses. 2023 Dec 19;16(1):6. doi: 10.3390/v16010006 (PMC10819642; doi:10.3390/v16010006)
Supplement: Supplementary file 1 [file viruses-16-00006-s001.zip › viruses-2718603-supplementary.pdf]

Supplementary Materials

Table S1. Demographic and clinical data of SMI patients. Informative clinical data about years of the disease progression.

Disease		N	Age (±SD)	Years of disease
SMI patients				
	Male	32	53.9 (±6.12)	23.5(±12.7)
	Female	39	54.7 (±8.04)	20.9(±11.7)
***P*-value**				**0.432**
